# Neutrophil elastase inhibitor purification strategy from cowpea seeds

**DOI:** 10.1371/journal.pone.0223713

**Published:** 2019-10-10

**Authors:** Graziele Cristina Ferreira, Adriana Feliciano Alves Duran, Flavia Ribeiro Santos da Silva, Livia de Moraes Bomediano, Gabriel Capella Machado, Sergio Daishi Sasaki

**Affiliations:** Centro de Ciências Naturais e Humanas, Universidade Federal do ABC, São Bernardo do Campo, São Paulo, Brazil; Instituto Butantan, BRAZIL

## Abstract

Serine proteases and its inhibitors are involved in physiological process and its deregulation lead to various diseases like Chronic Obstructive Pulmonary Disease (COPD), pulmonary emphysema, skin diseases, atherosclerosis, coagulation diseases, cancer, inflammatory diseases, neuronal disorders and other diseases. Serine protease inhibitors have been described in many species, as well as in plants, including cowpea beans (*Vigna unguiculata* (L.) Walp). Here, we purified and characterized a protease inhibitor, named VuEI (*Vigna unguiculata* elastase inhibitor), from *Vigna unguiculata*, with inhibitory activity against HNE (human neutrophil elastase) and chymotrypsin but has no inhibitory activity against trypsin and thrombin. VuEI was obtained by alkaline protein extraction followed by three different chromatographic steps in sequence. First, an ion exchange chromatography using Hitrap Q column was employed, followed by two reversed-phase chromatography using Source15RPC and ACE18 columns. The molecular mass of VuEI was estimated in 10.99 kDa by MALDI-TOF mass spectrometry. The dissociation constant (Ki) to HNE was 9 pM. These data indicate that VuEI is a potent inhibitor of human neutrophil elastase, besides to inhibit chymotrypsin.

## 1. Introduction

Protease inhibitors (PIs) are regulatory proteins that control proteolytic events in living organism [1] present in different tissues of animals, plants and microorganisms. [2]. There are different mechanisms of protease inhibition. Some type of inhibitors form reversible stoichiometric protein-protein complexes with their target proteolytic enzymes, like Bowman-Birk (BBI) (I12) and Kunitz (I3) from plants [3, 4] while others PIs form irreversible complexes with their target enzymes, like serpins [5].

Plant PIs are involved in an impressive variety of biological processes [6], and may form part of plant-based defense processes, as well as part of complex resistance activation cascades [7].

Serine proteases inhibitors have been described in plants, six families of these inhibitors presents characterized members in plants (I3, I6, I7, I12, I18 and I20), the families I3 (Kunitz) and I12 (BBI) are the most studied, they are able to inactivate several enzymes with variable degrees of specificity [4, 8–10].

The first BBI characterized was isolated from soybean (*Glycine max*) [11, 12] being the most studied member and considered the standard BBI [13, 14]. BBIs are highly stable proteins containing 6 to 7 disulfide bonds which contribute to their stability. BBI have molecular mass around 7 to 10 kDa and two reactive sites; usually the first reactive site inhibits trypsin, while the second inhibits other different proteases. The two domains are exposed and are readily accessible to proteolytic enzymes [9, 15, 16]. Most legume inhibitors present activity against serine proteases by the canonical mechanism [17]. BBIs of different dicotyledons present high amino acid sequence homology [18], likely related to the interaction between BBIs and proteases that have a coevolution relationship, as related in insects [19]. In general, legume seeds contain multiple molecular forms of BBI that may arise due to post-translational modifications, such as formation of hydrogen bonds [20, 21].

Plant Kunitz inhibitors are proteins with a molecular weight of 18 to 22 kDa, which contain two disulfide bonds formed by cysteines and commonly contain only one reactive site [9]. Studies have shown that Kunitz inhibitors may be active against trypsin, chymotrypsin and elastase [22]. In plants, a prominent family in Kunitz is the I3, which encompasses the I3A subfamily with a widely studied representative named Kunitz Soybean Trypsin Inhibitor (SBTI), inhibitor of trypsin and chymotrypsin present in soybean (*Glycine max*). Members from this subfamily present conserved residues that play a role in the inhibition efficacy, stabilizing the canonical bond that surrounds the reactive site [13, 23, 24]. The I3 family, primarily composed by inhibitors from Leguminosae (legumes), usually exhibits a polypeptide chain, one reactive site and is capable of inhibiting serine proteases, cysteine proteases and aspartyl protease. The major number of inhibitors from this family have two disulfide bonds, but they may present a variable number of cysteines and consequent variation in the number of disulfide bridges [13, 23, 25]. The reactive site of the Kunitz trypsin inhibitors usually exhibits amino acids Lys or Arg and in the case of elastase inhibitors, this position is occupied by an Ala residue, with possible variation [26, 27].

Black-Eyed Pea Trypsin Chymotrypsin Inhibitor (BTCI) is one of the most described BBI, present in seeds of *Vigna unguiculata*. The inhibitor shows inhibition constant (Ki) 14 nM for trypsin and 7.6 nM for chymotrypsin [28] with 83 residues and seven disulfide bonds [29], may be this disulfide bond responsible for the remarkable stability exhibited by this inhibitor [30]. It has molecular weight of 9.07 kDa and shows high pH and temperature stability [30], and crystallization of the inhibitor with trypsin was carried out by Barbosa and collaborators [31]. BTCI is able to inhibit trypsin and chymotrypsin, and its reactive sites act independently [32]. There are studies that indicate that BTCI has anticancer potential [33] and suppresses human breast adenocarcinoma cell viability [34].

Additional inhibitors were purified in *Vigna unguiculata*, using different processes such as CpTI trypsin inhibitor, purified by ammonium sulfate precipitation, followed by anion-exchange chromatography (Super-Q) and gel filtration (Sephadex G-200). CpTI has approximately 8 kDa and thermal stability (up to 90°C) [35]. Another inhibitor identified in the *Vigna unguiculata* is the CpPI (in two cowpea cultivars: Cream7 and Buff), purified by ammonium sulfate fractionation and DEAE-Sephadex A-25 column. It has been demonstrated that CpPI is involved in the insect development of *Spodoptera littoralis* [36]. Inhibitors with similar characteristics were purified in the cultivars Cream7 and Buff with sizes of 17.1 kDa and 16.5 kDa, Ki for trypsin 0.53 nM and 0.436 nM and chymotrypsin 0.99 nM and 0.10 nM, respectively. To our knowledge there are no reports regarding neutrophil elastase inhibition activity by BTCI or any other protease inhibitor in *Vigna unguiculata*, while in other species of the same genus inhibitors have already been characterized.

On the other hand, in Vigna genus, inhibitors to chymotrypsin, subtilisin and aspartic protease have already been characterized. In *Vigna mungo*, three different isoforms of Black gram Trypsin Inhibitor (BgTI), of 16 kDa, can inhibit trypsin and chymotrypsin, each isoform presents different Ki for each protease, but all on nM scale. These inhibitors were purified by SP-Sepharose, Q-Sepharose, Mono Q and Mono S, and Superdex 75. Another inhibitor in the same specie BgPI inhibits trypsin and chymotrypsin and has high structural stability; this 8 kDa BBI was purified by ammonium sulfate prepping followed by 3 chromatography steps [37]. Adzuki Subtilisin Inhibitor (ASI), an inhibitor purified from *Vigna angularis*, can inhibit subtilisin with a Ki of 0.16 nM. The 10.8 kDa molecule was identified as being from the PotatoI family through amino acid sequence analysis, and was purified by CM-cellulose, Sephadex G-75, DEAE-Cellulose and Sephadex C-25 [38, 39]. Plant inhibitors do not inhibit only serine proteases, in *Vigna radiate* an inhibitor of aspartic protease (VrAP) of 16.6 kDa with Ki in nM range was characterized. Its purification was performed by precipitating acid filtration and followed by reverse phase chromatography [40].

Considering the importance of human neutrophil elastase (HNE) in several biological processes and its involvement in inflammatory processes and immune response, this work investigated the presence of HNE inhibitors in *Vigna unguiculata* seed extract.

## 2. Materials and methods

### 2.1. Materials

Cowpea (*Vigna unguiculata* (L.) Walp) seeds of the cultivar BRS Marataoã, generated by genetic improvement, were supplied by the “EMBRAPA MEIO NORTE”, Teresina, PI, Brazil. All the substrates employed were supplied by Calbiochem®, as follow: Fluorogenic V (MeO-Suc-Ala-Ala-Pro-Val-AMC) CAS number 72252-90-5; Z-Phe-Arg 7-amido-4-methylcoumarin hydrochloride, CAS number 65147-22-0; Chymotrypsin Substrate II (Suc-Ala-Ala-Pro-Phe-AMC), CAS 88467-45-2; N-Benzoyl-Phe-Val-Arg-7-amido-4-methylcoumarin, CAS number 88899-22-3. The enzymes used were: Human Neutrophil Elastase (HNE) (EC 3.4.21.37), Calbiochem CAS number 9004-06-2, Trypsin from bovine pancreas (Sigma-Aldrich) (EC 3.4.21.4), CAS number 9002-07-7; α-chymotrypsin from bovine pancreas (EC 3.4.21.1), Sigma-Aldrich, CAS number 9004-07-3; Thrombin from bovine plasma (EC 3.4.21.5) Calbiochem®, CAS number 9002-04-4.

### 2.2 Protein extraction

Alkaline extraction was performed using 5 g of beans in each preparation. The cowpea was shredded in the high-speed stainless steel blender Skymsen. To the obtained material was added 100 mM Tris-HCl pH 8.0 in the proportion 1:10 (m/v), with stirring for 2 hours at 12°C. After stirring the residue was filtered with sterile gauze to remove the solid residue from the supernatant, then the supernatant was centrifuged at 6603 RCF for 30 minutes at 4°C and the supernatant was collected. The samples were again centrifuged at 14167 RCF for 20 minutes at 4°C, followed by a filtration process with a vacuum filtration system, Sartolab® 0.22 μm membrane. The obtained material was stored at -20°C.

### 2.3 Protein purification

The supernatant of the extract from item 2.2 was diluted 1:1 with autoclaved ultrapure water and subjected to sequential processes of different purifications. The ion- exchange chromatography was initially performed using a 5 mL (GE®) HiTrap Q-FF column with ÄKTA prime plus (GE®) equipment. It was applied 10 mL of the protein extract sample (previous diluted in 1:1 in water) into the Hitrap-Q column. The protein elution were performed on a 0–1 M NaCl linear gradient using 100 mM Tris-HCl buffer (pH 8.0), and fractions of 2 mL were collected. The Ion-exchange chromatography was repeated ten times. The samples from Ion-exchange chromatographies, that presented inhibitory activity toward neutrophil elastase, were pooled and concentrated using Centrivap Concentrator (LABCONCO), a final sample of 3 mL were obtained and 1 mL were applied in the second chromatographic step. The samples were chromatographed in the reverse-phase SOURCE 15RPC ST 4.6 / 100 column (GE Healthcare) using ÄKTA Purifier 10 (GE Healthcare) equipment. The elution were performed with linear gradient of 0–100% of acetonitrile with 0.050% TFA, fractions of 500 μL were collected. The eluted fractions from reverse-phase chromatography had the acetonitrile removed by evaporation using a Centrivap Vaccum Concentrator. These samples, after total removal of acetonitrile were solubilized in 100 μL of deionized water and tested toward human neutrophil elastase to find the inhibitory activity. This step was reproduced three times. The isolated peaks that presented inhibitory activity toward HNE, from the previous stage, were subjected to reverse phase chromatography with an ACE 5 C 18–300 column (ACE). The column was equilibrated with 0.065% TFA in ultrapure water and proteins were eluted in 500 μl fractions with a gradient of 0–100% of acetonitrile with 0.050% TFA, the eluted fractions had the acetonitrile removed by evaporation using a Centrivap Vaccum Concentrator, the samples were solubilized in 100 μL of deionized water and tested toward different proteases.

### 2.4 Determination of protein concentration

Total protein content was measured as described by Bradford [41] with bovine serum albumin as the standard protein and using the Bio-Rad Protein Assay® kit with 595 nm absorption readings on plate spectrophotometer (Synergy HT (Biotek)® multi-mode microplate reader).

### 2.5 SDS-PAGE

SDS-PAGE without reducing agent was carried out according to the method of Laemmli [42] using 10% or 12% polyacrylamide gels with sample Buffer (0.25 M Tris-HCl pH 6.80, 10% SDS, 50% Glycerol and 0.5% bromophenol blue). The molecular weight marker used was the Prestained Broad Range SDS-PAGE Standards (BIO-RAD®). The gel was stained by the addition of Bio-Safe Coomassie G-250 Stain (BIO-RAD®).

### 2.6 Serine protease inhibition assays

The inhibitor activity was measured (during 20 minutes) by the remaining hydrolytic activity of the enzymes on the substrate at pH 8.0 after pre-incubation with inhibitor samples for 10 minutes at 37 ^o^C [43]. The active HNE (Calbiochem, San Diego, CA, USA) concentration was determined using alpha1-antitrypsin previously titrated with active trypsin. The trypsin active site titration was performed with p-nitrophenyl-p-guanidine-benzoate (Sigma, St. Louis, MO, USA) as described by Chase and Shaw (1969) [44].

All inhibitory assays were performed using 100 mM Tris-HCl buffer (pH 8.0) containing 0.15 M NaCl and 0.1% Triton X-100 at 37°C. The fluorescence readings were obtained using the Synergy HT Biotek multi-mode microplate reader. The used wavelengths were 320/20 (excitation) and 460/40 (emission). The substrates used were: Fluorogenic substrate V (MeO-Suc-Ala-Ala-Pro-Val-AMC) (0.08 mM), Z-Phe-Arg-AMC (0.04 mM), Ala-Ala-Pro-Phe-AMC (0.08 mM), and N-Benzoyl-Phe-Val-Arg-AMC (0.04 mM), for measure the activity of HNE, bovine Trypsin, chymotrypsin and bovine thrombin, respectively. The proteases were used in the follow final concentration: HNE (1 μg/mL), Bovine trypsin (0.25 μg/mL), bovine pancreas alpha-chymotrypsin (0.25 μg/mL) and Bovine Thrombin (0.25 μg/mL). Chymotrypsin and bovine thrombin had their concentration determined by dilution of known amount of each enzyme in assay buffer. For screening of inhibitory activity toward HNE along the purification process were used 100 μL of each 2 mL sample from Hitrap-Q column; and 10 μL of each 100 μL sample from Source 15 RPC column or ACE 18 column, after concentration and dilution in deionized water. To determine the inhibitory activity of purified VuEI toward different proteases were used 5.1 μg of the inhibitor in the assays.

We clarify the origin of each substrate and enzyme in the item 2.1. The quantity of protein in each sample was included in the results.

### 2.7 Ki determination

The partially purified sample had the active inhibitor concentration and the inhibitory Ki constant determined by the residual activity of the HNE enzyme, using increasing concentrations of the inhibitor. Apparent Ki values were calculated by fitting the steady-state velocities into the equation (*V*_*i*_/*V*_*o*_ = 1 − {*E*_*t*_ + *I*_*t*_ + *K*_*i*_ − [(*E*_*t*_ + *I*_*t*_ + *K*_*i*_)^2^ − 4*E*_*t*_*I*_*t*_]^1/2^}/2*E*_*t*_) for tight-binding inhibitors and using a non-linear regression analysis to [45] using the Grafit software (version 3.01).

### 2.8 Mass spectrometry

The sample containing VuEI (16 pmol) was spotted onto a MSP 96 polished steel target plate (Bruker Daltonics, Germany) and air-dried at room temperature. The spots were covered with 2 μL of saturated matrix solution (alpha-cyano-4-hydroxycinnamic acid, Bruker Daltonics, Germany) prepared in 50% acetonitrile HPLC Plus (Sigma Aldrich) and 0.1% trifluoroacetic acid (Pierce Biotechnology, USA). The mass spectra were acquired using MALDI-TOF MS spectrometer in a linear positive mode (Microflex, Bruker Daltonics, Germany). Bacterial test standard (BTS, Bruker) was used for instrument calibration. The mass spectra data was analyzed in a range of 2,000 to 20,000 m/z.

## 3. Results and discussion

### 3.1 Protein extraction

**Fig 1:**
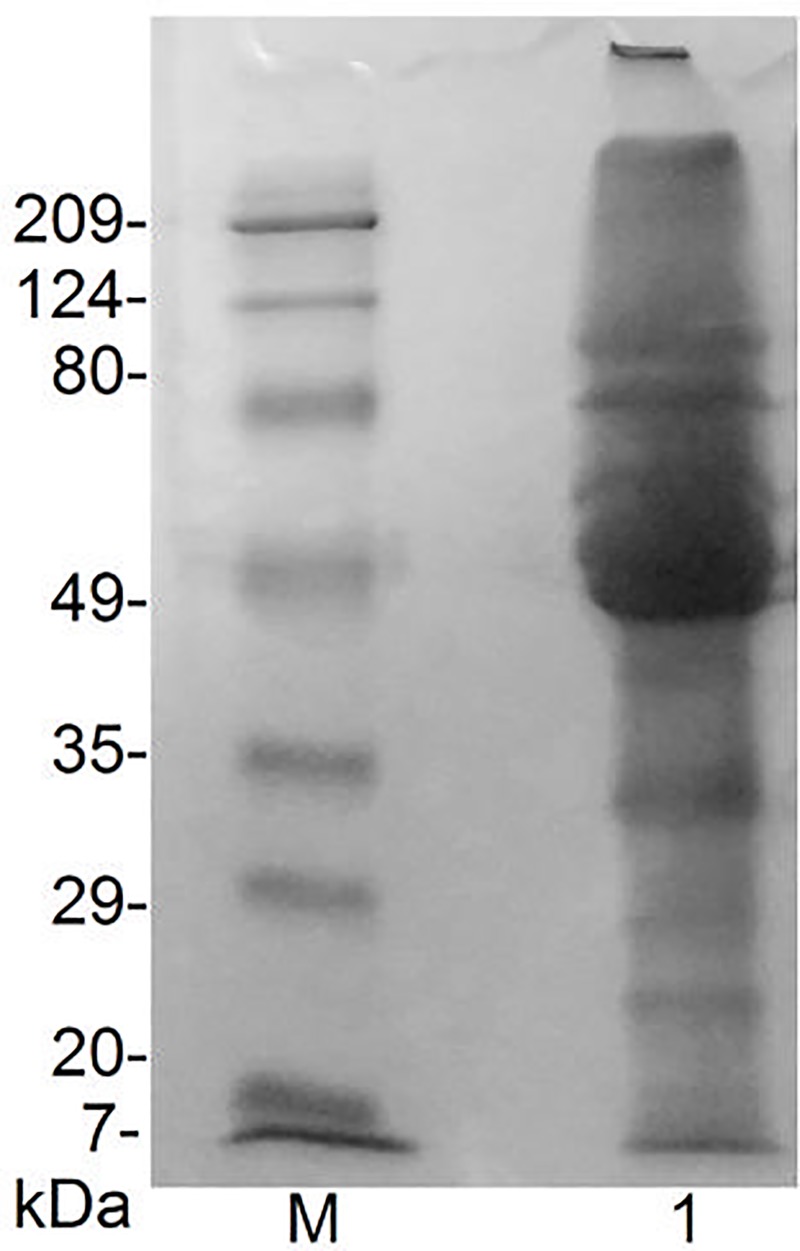
SDS-PAGE of *Vigna unguiculata* protein extract. The separation gel used was at 10%. M—Prestained Broad Range molecular weight marker SDS-PAGE standard BIO-RAD® (5 μL), Lane 1: *Vigna unguiculata* protein extract in non-reducing condition.

Total protein of 102.30 mg was extracted from 5 g of seeds in Tris-HCl buffer and 10 μg was applied on SDS-PAGE. Proteins of sizes ranging from 7 kDa to more than 200 kDa were observed as shown in Fig 1. The higher sizes proteins likely correspond to globulins and albumins, as previously shown in the literature about legumes. Albumins are water soluble and comprise enzymatic proteins, protease inhibitors, amylase inhibitors and lectins and have molecular masses (MM) ranging between 5 kDa and 80 kDa. Globulins represent roughly 70% of legume seed proteins, and their molecular masses range from 8 to 600 kDa [46]. It has been previously shown by Chan and Phillips, that 66% of the protein fraction of *Vigna unguiculata* demonstrated a fraction of globulins with polypeptides in a range of 65–50 kDa, albumins representing 24.9% of the total, with proteins between 30–99 kDa, and proteins present in lower concentration, glutelin 4.7% presenting 44–62 kDa and prolamins 0.7% with proteins of 54–105 kDa [47].

Our analysis of *Vigna unguiculata* protein extract showed proteins between 30 kDa and 7 kDa, the range we expected to find inhibitors, in concordance with masses of Kunitz (18 kDa to 22 kDa) and/or BBI (7 kDa to 9 kDa) inhibitor families. On the other hand, protease inhibitors can aggregate and form highly stable dimers in solution, increasing their molecular masses [48]. Considering this possibility, as the SDS-PAGE was carried out under non-reducing conditions it is possible that part of the proteins (larger than 30 kDa) observed in the SDS-PAGE can be protease inhibitors aggregated.

### 3.2 Purification

An HNE inhibitor molecule was purified by three different chromatographic steps. The fractions eluted by each chromatography which showed inhibitory activity to HNE were pooled and used in the subsequent steps and after 3 processes we obtained the purified protein. The protein profile of the samples can be verified through SDS-PAGE.

**Fig 2:**
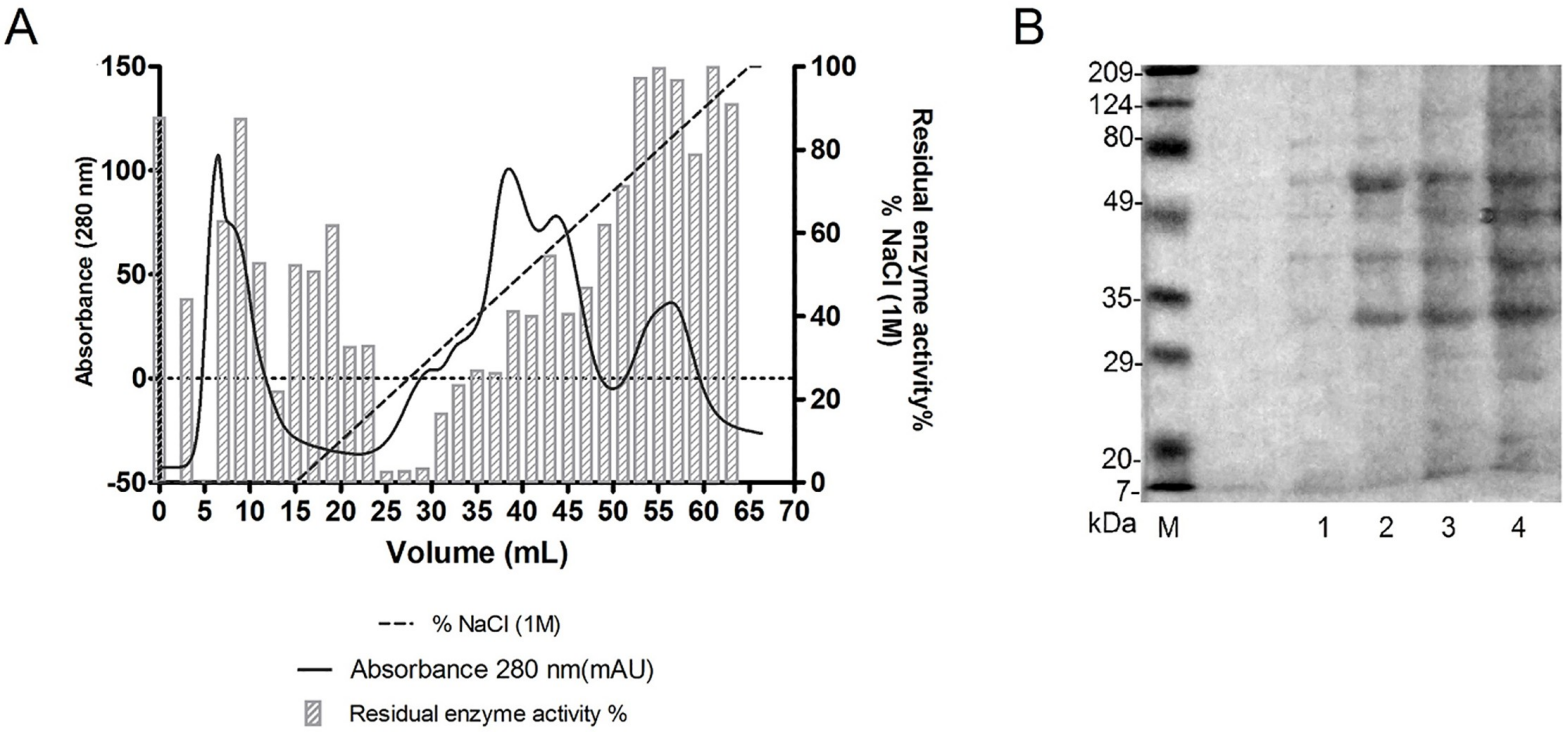
Ion-Exchange chromatography of *Vigna unguiculata* protein extract using HitrapQ (GE-Healthcare) column. **A)** Chromatogram. Columns indicate the residual activity of the neutrophil elastase enzyme (scale on the right y axis) in assays using the purified fractions (x axis). **B)** SDS-PAGE of samples obtained from Hitrap-Q column chromatography that showed inhibitory activity on HNE, it was used a 10% separation gel. M—Prestained Broad Range Molecular Marker (BIO-RAD); Lane 1: Fraction corresponding to 23–24 mL; Lane 2: Fraction corresponding to 25–26 mL; Lane 3: fraction corresponding to 27–28 mL; Lane 4: fraction corresponding to 29–30 mL.

In the first stage of purification, an Ion-Exchange chromatography with Hitrap-Q (GE-Healthcare) column was used. Elution fractions between 24 mL and 30 mL presented the highest inhibitory activity against HNE (Fig 2A). However as shown in Fig 2B, proteins of different sizes are observed in this sample

The samples from Ion-exchange chromatography with inhibitory activity toward neutrophil elastase (23–30 mL elution samples) were concentrated using Centrivap Concentrator (LABCONCO®) and applied in the second chromatographic step.

**Fig 3:**
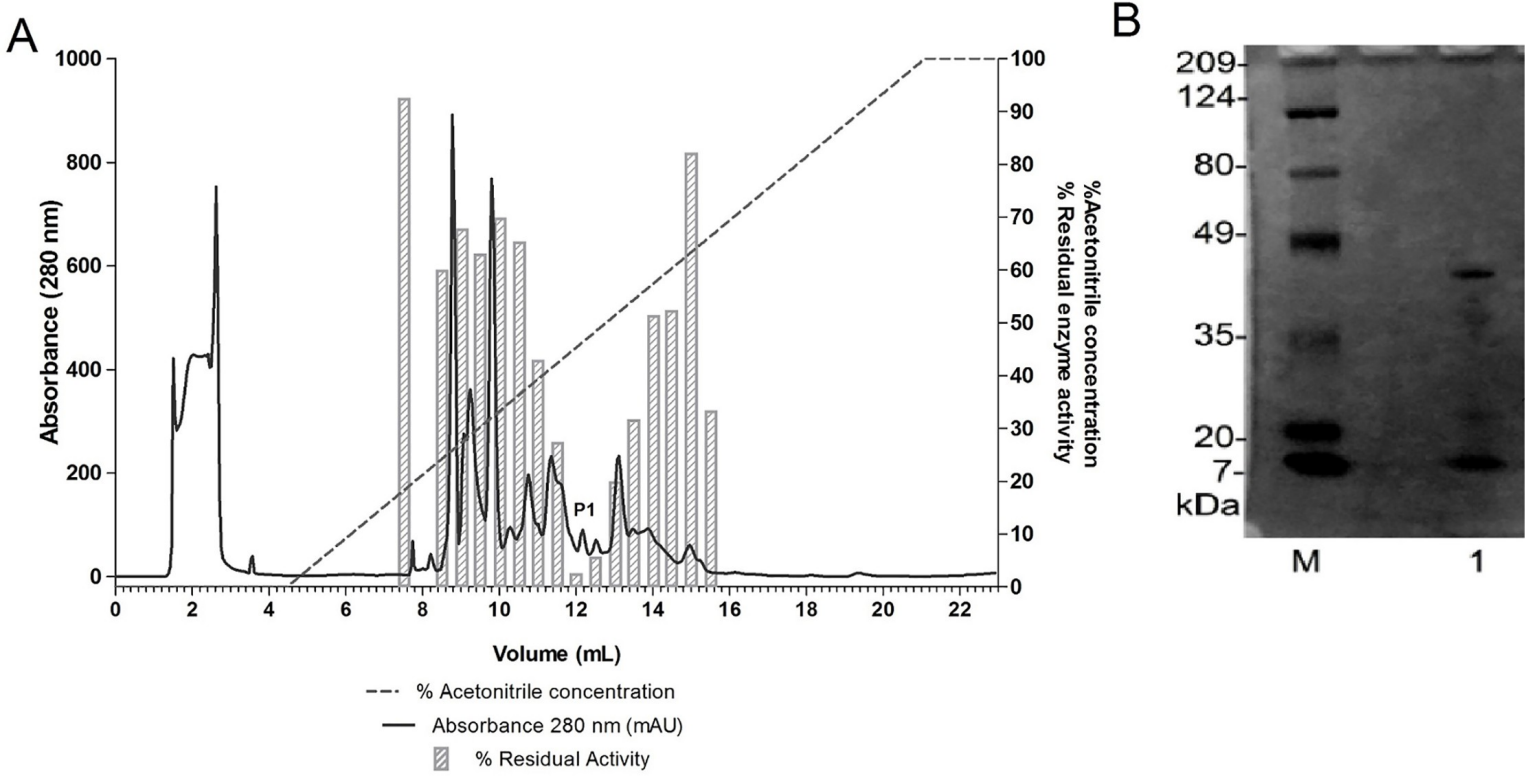
Second purification step using reverse-phase chromatography on 15RPC column. **A)** Chromatogram obtained using Source 15RPC (GE-healthcare) column. It was applied the protein pool purified by Ion-exchange chromatography with HNE inhibitory activity. Columns indicate the residual activity of the HNE (scale on the right y axis) in assays using the purified fractions (x axis). Sample with highest HNE inhibitory activity was named P1. **B)** SDS-PAGE (10%) of the samples from reverse phase chromatography with the SOURCE 15RPC column which showed inhibitory activity. M- Prestained Broad Range Molecular Marker (BIO-RAD); Lane 1: Fraction corresponding to elution in 12 mL, named P1.

In the second step of the purification, a reverse phase chromatography using Source 15RPC (GE-healthcare) column was used to further separate the proteins with inhibitory activity, achieving the isolation of a unique inhibitor. Fraction P1, corresponding the fraction eluted around 12 mL, presented the highest inhibitory activity (Fig 3A) and its SDS-PAGE profile shows that at least three major proteins in intensity are present in that sample; however other minor proteins are also present in the sample.

**Fig 4:**
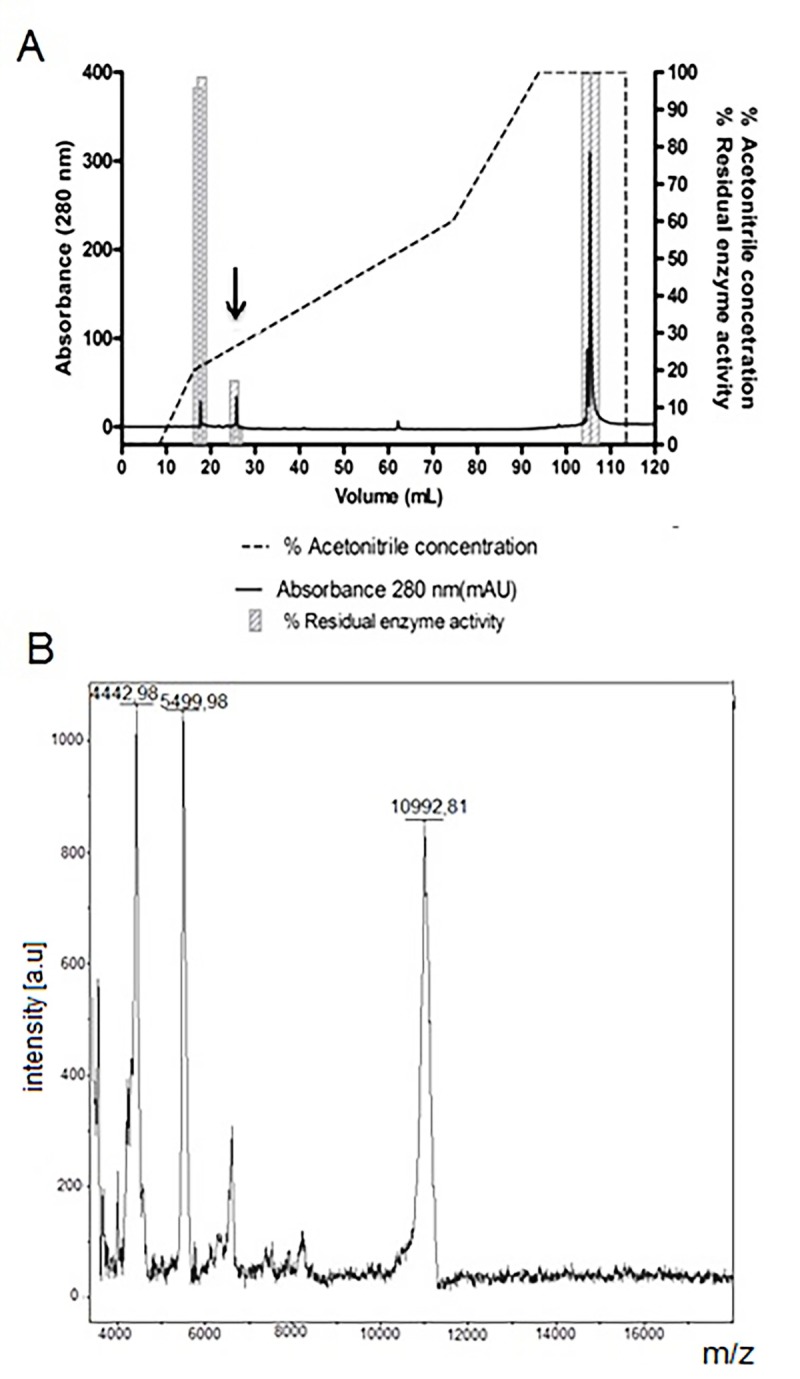
Third purification step using reverse-phase chromatography on ACE18column. **A)** Chromatogram obtained using ACE18 (ACE) column. It was applied the P1 fraction purified by Source 15RPC column. Columns indicate the residual activity of the HNE (scale on the right y axis) in assays using the purified fractions (x axis). The arrow in A indicates the sample with inhibitory activity toward HNE. **B)** Mass spectrometry—MALDI-TOF of the sample indicated by arrow in A.

In the last purification step, a reverse-phase chromatography employing ACE18 (ACE) column was used, to separate the proteins observed in P1 fraction obtained previously. The chromatogram in Fig 4A shows three peaks of UV absorbance. These samples were tested to HNE to check the inhibitory activity. The first peak was eluted in 17 mL to 18 mL, corresponding to 96% and 99% of residual enzyme activity of HNE, respectively (indicating no inhibition). The second peak corresponds to elution in the volume of 25.5 mL to 26 mL, presenting 17.3% and 5.4% residual enzyme activity of HNE (inhibition of 82.7% and 94.6%). This UV peak corresponds to the serine protease inhibitor described in this work. Finally, the third peak represents two fractions corresponding to 104.5 mL to 106 mL they present 100% of residual enzyme activity of HNE (no inhibition). These results indicate that the first and the third UV peaks didn´t present inhibitory activity for HNE, so according to the inhibitory activity screening toward HNE, we can verify that the inhibitory molecule is in the second protein peak of the chromatography, eluted around 26 mL. The MALDI-TOF mass spectrometry (Fig 4B) shows a protein with a molecular mass of 10.99 kDa; considering the majority peak as the masses presented by BBI family, one of two families that have already been described in legume, we suggest that the purified inhibitor belongs to BBI family. This inhibitor was named *Vigna unguiculata* elastase inhibitor, abbreviated as VuEI. The other two peaks observed in the figure are the peak of double ionization of VuEI (5499 Da) and a co-purified protein with a lower molecular mass (4442 Da). However, due the close yield value obtained in the purification process (Table 1), comparing the sample from Source 15 RPC and ACE 18 columns we assumed that the co-purified protein does not interfere with VuEI activity.

**Table 1:** Purification steps and yield of VuEI from the cowpea extract.

Purification step	Total Protein (mg)	Inhibitory Units (U)	Specific Activity (U/μg)	Fold	Yield%
**Extract**	102.3	295	2.88	1	100
**Hitrap Q FF**	7.8	408	52.31	18.1	138
**Source 15 RPC**	0.283	4.66	16.44	5.7	1.5
**ACE 18**	0.011	5.72	511.63	177.4	1.9

Inhibitory Units: One inhibitory unit was defined as the quantity of inhibitor that inhibits 1 μg of elastase. Specific Activity was calculated by: Inhibitory Units/Total Protein in each purification step. Fold was calculated by ratio between Specific Activity of each purification step and Specific Activity of the Extract. Yield%: was determined by the ratio between Inhibitory Units of each purification step and Inhibitory Units of the Extract, the values were presented in percentage mode.

Regarding the purification process, we can observe in Table 1 the amount of protein contained in pools of samples that presented inhibitory activity toward HNE in each purification step. From 5 g of seed mass we obtained 102.3 mg of protein extract and after the purification steps, 11 μg of the purified inhibitor.

Previous purification of other neutrophil elastase inhibitors was carried out using different methodologies in several plant species obtaining different yields. *Caesalpinia echinata* elastase inhibitor (CeEI), belonging to the Kunitz family, was purified from the *Caesalpinia echinata* seeds extract by ion exchange chromatography (Resource Q column) followed by hydrophobic interaction chromatography (Octyl Fast Flow column), and reverse phase chromatography (CLC-ODS Shim Pack column). CeEI was able to inhibit neutrophil elastase and cathepsin G at a nanomolar range (with Kis of 1.9 and 3.6 nM, respectively) and inhibited PR3 within a micromolar range (Ki 3.7 μM) [49]. *Bauhinia bauhinioides* Cruzipain Inhibitor (BbCI), belonging to Kunitz family too, was obtained from *Bauhinia bauhinioides* seeds. BbCI was purified by ion exchange chromatography using DEAE-Sephadex column, followed by affinity chromatography (trypsin-Sepharose column), ion exchange chromatography (Mono Q column) and reverse-phase chromatography (C4 column). BbCI presents inhibitory activity against cruzipain with dissociation constant (Ki) of 1.2 nM, cruzain (Ki 0.3 nM), a C-terminally truncated recombinant species of cruzipain, and Cathepsin L is also inhibited (Ki 0.22 nM) [50]. The recombinant form of BbCI inhibits human neutrophil elastase (Ki(app) 5.3 nM), porcine pancreatic elastase (Ki(app) 40 nM), cathepsin G (Ki(app) 160 nM) and the cysteine proteinases cruzipain (Ki(app) 1.2 nM), cruzain (Ki(app) 0.3 nM) and cathepsin L (Ki(app) 2.2 nM) [51]. VuEI is more specific to inhibit neutrophil elastase compared with CeEI and BbCI. VuEI presents a Ki to neutrophil elastase of 9 pM lower than Ki presented by CeEI and BbCI. For both inhibitors, CeEI and BbCI were not possible to compare the purification processes yield, because the authors did not published the purification data. However, some purification data is available for trypsin inhibitors from other Vigna species. From *Vigna radiata* (L.) R. Wilczek, the seeds were processed by heat-treatment, followed by ammonium sulphate precipitation and gel filtration on Sephadex G-50, in this case, the inhibitor yield after the last purification step was 30.25% and the purity fold was 13.51 [52], they obtained higher yield and lower purity fold value compared with VuEI purification (Table 1). In *Vigna mungo*, three isoinhibitors named Black gram trypsin inhibitor (BGTI) with 16 kDa that inhibit trypsin and chymotrypsin, were purified by SP-Sepharose, Q-Sepharose, Mono Q and Mono S, and Superdex 75. Purification fold of BGTI1 was 20.9, and of BGTI2 and BGTI3 were 20.6 and 18.8, respectively. The isoinhibitor BGTI1 and BGTI2 had a yield of 2%, and BGT3 1% [53]. VuEI purification process presented fold value higher than BGTI isoinhibitors purification and lower yield than it (Table 1).

On the other hand, it was possible to compare the VuEI purification process with two BBI from plants, *Cratylia mollis* Trypsin Inhibitor (CmTI) from *Cratylia mollis* seeds [16] and *Torresea acreana* Trypsin Inhibitor (TaTI) from *Torresea acreana* seeds [14]. The purification method used to purify VuEI presented higher fold value, 177, in the last step of purification when compared with the purification procedures employed in purification of CmTI, 18 [16], however the yield in the CmTI purification was 30% higher than in VuEI method (1.9%). If we compare VuEI purification method with the purification of TaTI [14],as fold value as yield are higher in TaTI purification (fold = 1053; yield = 23) than in VuEI purification (fold = 177; yield = 1.9). CmTI and TaTI used the same purification method, the first step was a fractionation with acetone followed by two purification steps an ion-exchange chromatography using a DEAE-Sephacel column and a gel filtration chromatography using a Superdex 75 column. Our method was performed using as first step an ion-exchange chromatography followed by two reverse phase chromatographies steps.

In our analysis, the loss of inhibitory activity against neutrophil elastase occurred after the first reverse phase chromatography (using Source 15 RPC column), the Specific Activity obtained in this step was lower than the other purification steps (Table 1). Probably, the denaturation promoted by acetonitrile reduces VuEI activity. Comparing the total protein obtained to VuEI we obtained 11.18 μg from 102 mg of extract, CmTI purification obtained 19 mg from 1200 mg and TaTI purification obtained 6.15 mg from 27200 mg. In this case, VuEI purification was similar to the TaTI purification, from the initial quantity of total protein were obtained 0.011% and 0.023% of the purified protein (after last step) to VuEI and TaTI, respectively.

### 3.3. Inhibitory activity

**Fig 5:**
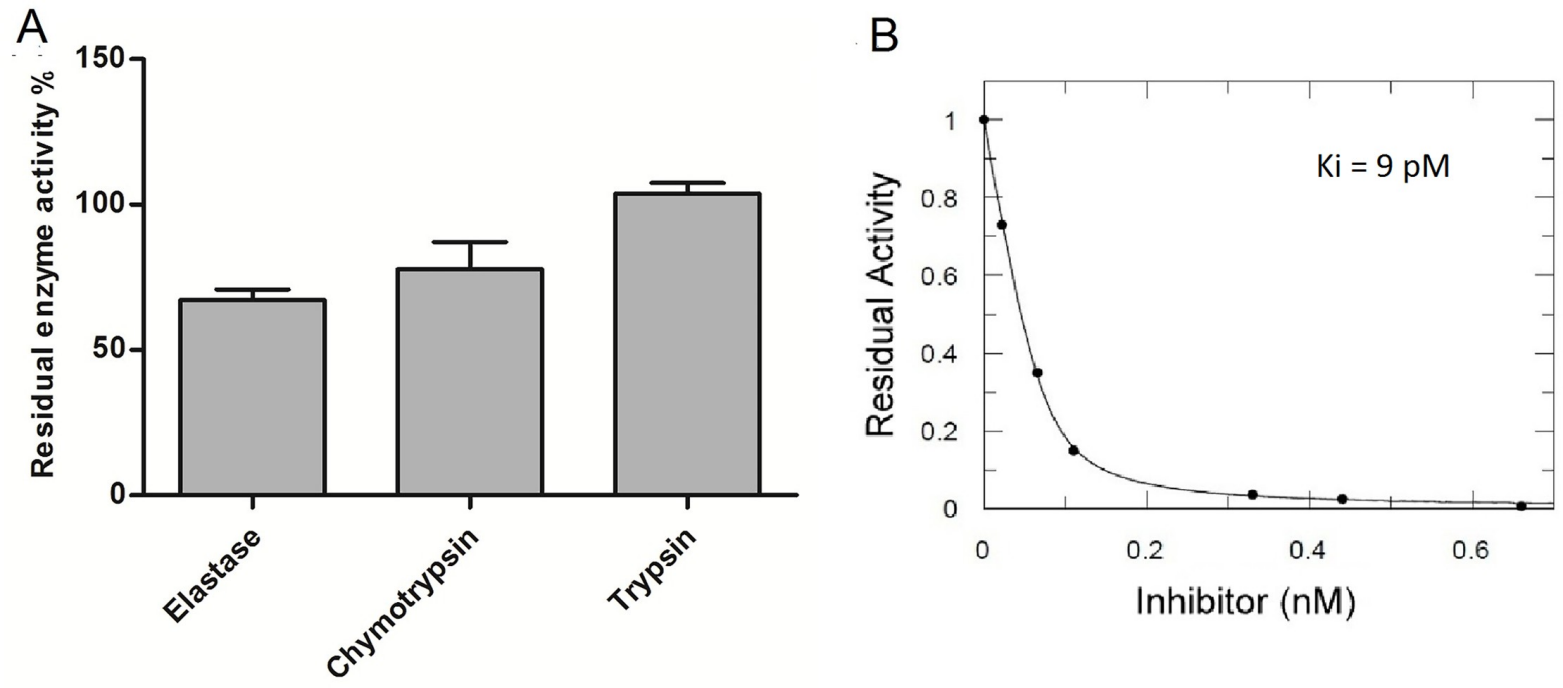
Enzymatic inhibitory assays using *Vigna unguiculata* Elastase Inhibitor. **A)** Inhibitory activity of purified sample (8 ng of protein) from ACE18 column toward HNE (1 μg/mL), bovine Trypsin (0.25 μg/mL), chymotrypsin (0.25 μg/mL) and thrombin (0.25 μg/mL); **B)** Inhibitory activity curve of Source15 RPC P1 sample toward HNE with different concentrations of inhibitor.

In addition to the inhibitory activity assays performed throughout the purification process, the purified VuEI was tested for inhibitory activity against four serine protease enzymes. VuEI present inhibitory activity on HNE (67.1% of residual enzyme activity) and chymotrypsin (77%), and did not interfere in the activity of bovine trypsin (Fig 5A) and thrombin enzymes (data not shown).

The inhibitory activities of VuEI indicate that this inhibitor has two different reactive inhibitory sites, because chymotrypsin is an enzyme with high affinity to hydrophobic amino acids of large side chains, such as Trp, Phe and, Tyr in P1 position of the reactive site [54], and, neutrophil elastase has affinity, in P1 position, for hydrophobic amino acids of small side chains as Ala and Val [55]. On the other hand, the serine proteases trypsin and thrombin, that were not inhibited by VuEI, have affinity in P1 position for basic amino acids, such as Arg and Lys [54, 55]. We reported here for the first time an inhibitor from legume that has activity toward chymotrypsin and neutrophil elastase and did not inhibit trypsin.

The fact that VuEI inhibits two different enzymes, with different preferential amino acid in P1 position of the reactive site, combined with the molecular mas determined by MALDI-TOF-MS (10.99 kDa) reinforce the hypothesis that it belongs to the BBI family, since normally these inhibitors present molecular masses around 7 kDa to 10 kDa and two distinct reactive sites [9, 16]

### 3.4. Determination of Ki

We performed the inhibitory assay to Ki determination toward HNE using the pre-purified sample from the first reverse phase chromatography step (Source 15 RPC column). The Ki determined was 9 pM, showing that VuEI is a specific and potent inhibitor to HNE (Fig 5B).

Based on our results, we can propose that VuEI presents two inhibitory active sites, one with a specific P1 position to HNE and another one for chymotrypsin. Additionally, as the Ki determined for HNE was 9 pM, VuEI is a potent inhibitor for HNE compared to other plant inhibitors, such as from *Glycine soja* (WSTI-IV and WSTI-V, (Wild Soja Trypsin Inhibitor)), *Caesalpinia echinata* (CeEI), *Bauhinia bauhinioides* (BbCI), *Lathyrus sativus* and *Tylosema escullentum*, which show dissociation constants on the nM scale [8, 49, 56–58].

Protease inhibitors are found in different legume seeds, such as soybean (*Glycine max* and *G*. *soja*), common bean (*Phaseolus vulgaris*), pea (*Pisum sativum*), grass pea (*Lathyrus sativus*) and lentils (*Lens culinaris*). In comparison with other plant families, legumes seeds are a rich source of BBI [15]. In *L*. *culinaris*, a trypsin/chymotrypsin inhibitor has been isolated and characterized [59]. In *Vigna unguiculata*, a BBI have already been described, BTCI, with inhibitory activity toward trypsin and chymotrypsin [32, 60]. Regarding the elastase inhibitors, so far two were founded in wild soybean (*G*. *soja*) protein extracts [57]. WSTI-IV and WSTI-V presented Ki values of 0.4 μM and 0.48 μM, respectively. Additionally, Ls_BBI_3c, an protease inhibitor from grass pea (*L*. *sativus*), inhibits, besides trypsin, elastase with a Ki value of 55 nM [56]. In comparison with these three inhibitors, VuEI inhibits elastase in lower Ki (9 pM), confirming its high capacity to inhibit elastase. Furthermore, VuEI is the first elastase inhibitor described in *Vigna unguiculata*.

The biological role of protease inhibitors in plants is still unclear. Questions about the occurrence of different types of inhibitors in different parts of a plant and the variation in their expression are not fully elucidated. However, it is assumed that these inhibitors could act as: 1) storage proteins, 2) endogenous protease regulators and 3) assisting in defense against pests and pathogens [61] with the ability to inhibit proteases from these invaders [1]. PIs have variable biological functions in legume seeds, and it has been described that they are involved in the mechanisms of seeds defense [10] and either in the physiological mechanisms during the development of the plant and the seed, preventing proteolysis during quiescence and germination [13, 23]. Genes encoding Kunitz type inhibitors from legumes has been used to transform transgenic crop varieties in order to develop resistance to insects [62]. There are many studies that seek the relationship between the inhibitors of serine proteases present in plants and the coevolution with microorganisms, insects, nematodes, birds and mammals that may be related to the defense mechanisms of plants and the ability of these organisms to adapt to these mechanisms [63, 64].

Digestive proteins are fundamental to the digestive process of plant predators and pests, so the effective action of these proteases is directly linked to their survival. Plant-herbivore coevolution has demonstrated that both partners have developed sophisticated mechanisms to overcome defenses elaborated by each other [65]. Protease inhibitors reduce the digestive efficiency of herbivores by inhibiting the digestive proteases of insects; in turn, insects can adapt to PIs, generally increasing protease levels, or expressing proteases that can´t be inhibited by the PI in question [66]. In one of these experiments protease activities in the larval midguts of the bruchids *Callosobruchus maculatus* and *Zabrotes subfasciatus* were investigated, with the growing of larva in *Vigna unguiculata* seeds. As results of this work, intestinal homogenates of larvae from *C*. *maculatus* and *Z*. *subfasciatus* suggest a larger amount of serine protease activities in *Z*. *subfasciatus* than in *C*. *maculatus*, the partial inhibition of these activities by aprotinin, suggest that these substrates are being hydrolyzed by serine proteases; the same work demonstrated that *C*. *maculatus* larvae contain more cysteine proteases than larvae of *Z*. *subfasciatus*. The complete inhibition of the enzymatic activities of midgut homogenates from larvae of *C*. *maculatus* and *Z*. *subfasciatus* with E-64 suggests the existence of cysteine proteinases in these species. The presence of a serine protease in intestinal homogenates from Z. *subfasciatus* was confirmed with synthetic substrates, the enzyme isolated was also capable of acting on the extended binding substrates used to assay chymotrypsin and elastase [67]. Based in the results presented by Silva and collaborators we speculate that VuEI has a physiological role to avoid the action of protease digestion by serine proteases produced by beans pests like *Z*. *subfasciatus*. Instead of the physiological role of VuEI in the plant, the inhibitor can also be explored as a molecule that can be the target of studies for pest control, antimicrobial protein that can be used in several health areas and inhibitor of proteases involved in clinical pathologies.

Although human neutrophil elastase (HNE) has an active role in many physiological processes in human organism, mainly involving innate immune response, tissue remodeling and inflammation, it is also associated with many disorders when its activity is deregulated [68–71]. According to the literature, HNE is involved with many inflammatory diseases such as COPD, cystic fibrosis, bronchiectasis, pulmonary arterial hypertension, pulmonary fibrosis, acute lung injury [68, 69, 72, 73], atherosclerosis, aortic aneurysm [74–76] and inflammatory renal diseases [77].

Development of synthetic inhibitors and natural inhibitors (protein inhibitors) of HNE as a therapeutic option has been in course. About synthetic inhibitors there are two main molecules tested Sivelestat and AZD9668. Sivelestat sodium hydrate is a synthetic highly specific and intravenously effective HNE inhibitor [78] developed and produced by Ono Pharmaceutical Co. Ltd [79]. Currently, its effects are under investigation and applied in medical trials for many diseases. Aikawa and collaborators investigated the effect of Sivelestat in patients with acute lung injury (ALI) associated with systemic inflammatory response syndrome (SIRS) receiving invasive mechanical ventilation. Patients who received Sivelestat could be free from mechanical ventilation faster than with those receiving conventional therapies without Sivelestat, they conclude that Sivelestat is clinically useful for treatment of ALI associated with SIRS [80]. Sivelestat appears to reduce HNE concentration and to improve pulmonary oxygenation in patients with ALI [81]. Sivelestat effects was also studied as therapeutically option in other scenarios, like preventing preserving antitumor immunity and reducing inflammatory processes in patients that undergo major surgeries [78, 82, 83]. The tests in cancer surgeries showed as effect a reduced surgical stress by decreasing the cytokine release by inhibition of the HNE activity and preserving antitumor immunity in patients after undergoing major cancer surgery [82]. Nomura et al. studied the effects of Sivelestat in patients with congenital heart disease and pulmonary hypertension who underwent surgery using bypass. The study concludes that administration of Sivelestat at the initiation of bypass surgery in the surgical treatment of neonates or infants who have congenital heart disease and pulmonary hypertension suppresses IL-8 and IL-10 [83]. All the studies and clinical trials cited concerning the use of Sivelestat as a therapeutic agent show the great potential this inhibitor has to prevent and treat HNE related diseases.

Other synthetic HNE inhibitor has been developed. AZD9668 is a selective inhibitor of HNE and has therapeutic potential for use to inflammatory lung diseases. Kuna et al. tested the efficacy and safety of AZD9668 versus placebo in patients with COPD treated with budesonide/formoterol and with a history of exacerbation. However, no short-term improvements in lung function, respiratory symptoms or exacerbation profile in COPD was found in the treated patients [84]. Moreover, Stockley et al demonstrated that AZD9668 has positive effect in patients with bronchiectasis. Patients were also separated in two groups, treated with AZD9668 and treated with the placebo for 4 weeks. The results showed that AZD9668 can improve lung function in patients with bronchiectasis and reduce sputum inflammatory biomarkers [85].

On the other hand, about the HNE natural inhibitors, two endogenous inhibitors have been used in treatments toward different diseases, Alpha-1-Antitrypsin (A1AT) and Elafin. Alpha-1-Antitrypsin is produced by hepatocytes, intestinal epithelial cells, neutrophils, pulmonary alveolar cells and macrophages. A1AT inhibits HNE and it is one of the major protein components circulating in plasma blood [86–88]. Clinical trials using A1AT as a therapeutical agent has been in course, especially for COPD, where the imbalance between A1AT and HNE is the major responsible for the development of the diseases [68–71]. Chapman et al. demonstrated the effect of the treatment with A1AT in patients with severe A1AT deficiency. The patients were separated in two groups, one treated with A1AT (180 patients) and other with placebo (87 patients), for 24 months. Results showed that the A1AT treated group had a decrease of 34% in the rate of lung density loss compared to placebo group. The study concluded that there is evidence that purified A1AT augmentation slows progression of emphysema [89]. Gaggar et al performed an experiment in patients with cystic fibrosis treated with inhaled A1AT. Analysis of the sputum of the patients treated with A1AT showed increased alpha1-antitrypsin levels dose dependent, meaning higher levels at the higher dose, this study suggest that A1AT is effectively and safely delivered in patients with cystic fibrosis, confirming the therapeutic action of the A1AT molecule [90]. As mentioned before, it is believed that A1AT augmentation can retard the progression of emphysema. Intravenous A1AT augmentation therapy has been in use for more than 20 years already and the results show that it is a safe procedure with positive consequences for treated patients [91].

Elafin is an endogenous serine protease inhibitor involved in many physiological processes and it has activity on HNE and proteinase-3 [92]. Elafin is associated with elastase and proteinase-3 control during inflammatory process. It also has antibacterial and antiviral activities [92]. Zaidi et al. conducted experiments with transgenic mice, which overexpressed human elafin in cardiovascular system, kidneys, lungs and skin. The results of these experiments showed evidence that elafin has positive effects in treating myocardial ischemia, viral inflammation and proliferative inflammatory vasculopathies suggesting that elafin action occurs mainly in the suppression of neutrophil-mediated inflammatory tissue damage [93].

Other natural serine protease inhibitors have already also been studied in disease models. Plant inhibitors like *Enterolobium contortisiliquum* trypsin inhibitor (EcTI) that is a polyspecific inhibitor of the Kunitz family, extracted from *Enterolobium contortisiliquum* seeds [94] and it has inhibitory activity on trypsin, chymotrypsin, plasma kallikrein, plasmin, and HNE with Ki in nM range [95]. EcTI were used in an experimental inflammatory model in C57/Bl6 mice induced by HNE, the results showed a decrease in pulmonary inflammation, oxidative stress and mechanical alterations, consolidating the therapeutic potential of the inhibitor [96]. *Bauhinia bauhinioides* cruzipain inhibitor (BbCI) is an 18 kDa Kunitz-type proteinase inhibitor isolated from *Bauhinia bauhinioides* seeds. BbCI inhibits the activity of different serine proteinases, such as human neutrophil elastase, porcine pancreatic elastase and cathepsin G. BbCI also inhibits the activity of cysteine proteinases, such as cathepsin L, cruzipain and cruzain [97]. In elastase-induced pulmonary emphysema model, C57/BL6 mice treated with inhibitor. BbCI improved lung mechanics, reduced lung inflammation and airspace enlargement and also decreased oxidative stress levels induced by elastase [98]. Additionally, CeEI, a HNE inhibitor from plant, has been tested in pulmonary edema model, presenting anti-inflammatory effects [49].

Finally, recombinant inhibitors from *Rhipicephalus (Boophilus) microplus* tick were tested, separately, in emphysema model in C57/BL6 mice. rBmTI-A and rBmTI-6 are Kunitz-BPTI inhibitors, rBmTI-A has inhibitory activity on HNE, trypsin, human plasma kallikrein and plasmin in nM range [99, 100], rBmTI-6 inhibit trypsin and plasmin [101]. Both inhibitors in mice model presented protective effect against emphysema development, also, the treatment using it was sufficient to reverse the loss of elastic recoil, the alveolar enlargement and the increase in the total number of cells in the bronchoalveolar lavage fluid (BALF), with a primary decrease in the number of macrophages. The prevention of emphysema development in the mice model, apparently, is related with a control of inflammatory response, due the inhibitory activities of rBmTI-A and rBmTI-6 [102–104].

## 4. Conclusion

As previously mentioned, serine protease HNE is directly involved in several respiratory diseases, such as COPD, other lung inflammations investigated in animal and cellular models and other inflammatory diseases, such as atherosclerosis, aortic aneurysm and inflammatory renal diseases. The study and development of HNE inhibitor with therapeutic potential is a good way to settle a treatment protocol to minimize the consequences of the mentioned diseases. As VuEI is a natural inhibitor such as EcTI, BbCI, CeEI, rBmTI-A and rBmTI-6, and has a high inhibitory activity against HNE (Ki = 9 pM) has the potential to be used to investigate its effect on the inflammatory diseases process of pulmonary emphysema and other inflammatory diseases using *in vitro* or *in vivo* tests.
